# Prediction and Testing of Biological Networks Underlying Intestinal Cancer

**DOI:** 10.1371/journal.pone.0012497

**Published:** 2010-09-01

**Authors:** Vishal N. Patel, Gurkan Bebek, John M. Mariadason, Donghai Wang, Leonard H. Augenlicht, Mark R. Chance

**Affiliations:** 1 Center for Proteomics and Bioinformatics, Case Western Reserve University, Cleveland, Ohio, United States of America; 2 Department of Genetics, Case Western Reserve University, Cleveland, Ohio, United States of America; 3 Case Comprehensive Cancer Center, Case Western Reserve University, Cleveland, Ohio, United States of America; 4 Department of Oncology, Montefiore Medical Center, Albert Einstein College of Medicine, Bronx, New York, United States of America; 5 Department of Physiology and Biophysics, Case Western Reserve University, Cleveland, Ohio, United States of America; Baylor College of Medicine, United States of America

## Abstract

Colorectal cancer progresses through an accumulation of somatic mutations, some of which reside in so-called “driver” genes that provide a growth advantage to the tumor. To identify points of intersection between driver gene pathways, we implemented a network analysis framework using protein interactions to predict likely connections – both precedented and novel – between key driver genes in cancer. We applied the framework to find significant connections between two genes, *Apc* and *Cdkn1a* (*p21*), known to be synergistic in tumorigenesis in mouse models. We then assessed the functional coherence of the resulting *Apc-Cdkn1a* network by engineering *in vivo* single node perturbations of the network: mouse models mutated individually at *Apc* (*Apc^1638N+/−^*) or *Cdkn1a* (*Cdkn1a^−/−^*), followed by measurements of protein and gene expression changes in intestinal epithelial tissue. We hypothesized that if the predicted network is biologically coherent (functional), then the predicted nodes should associate more specifically with dysregulated genes and proteins than stochastically selected genes and proteins. The predicted *Apc-Cdkn1a* network was significantly perturbed at the mRNA-level by both single gene knockouts, and the predictions were also strongly supported based on physical proximity and mRNA coexpression of proteomic targets. These results support the functional coherence of the proposed *Apc-Cdkn1a* network and also demonstrate how network-based predictions can be statistically tested using high-throughput biological data.

## Introduction

The majority of nonhereditary colorectal tumors arise via the sequential accumulation of mutations in key driver genes, where a mutation in a tumor suppressor (e.g. *Apc*) or oncogene (e.g. *Kras*) initiates the process, and a cascade of somatic mutations ensues [1]. Although these mutations were classically thought to be comprised of a few genes (e.g. *Apc*, *Kras*, *Trp53*), recent large-scale sequencing efforts revealed that any given tumor includes (on average) 80 mutations, with as many as 15 lying in frequently mutated “driver” genes [2]. In support of the hypothesis that these key genes function cooperatively in driving tumorigenesis, mouse models mutated at two driver genes simultaneously have shown a synergistic increase in tumor burden, including: *Pten-Apc* [3], *Kras-Tgfb* [4], and *Apc-Trp53* [5]. The evidence of synergistic, i.e. non-additive, increases in tumor burden suggest that the signaling pathways of two mutated genes may intersect downstream, and, thus, predicting and interrogating these points of intersection – ***as a biological network*** – is of significant interest. To trace the connections between genes, a variety of high-throughput datasets – e.g. protein-protein interactions (PPIs), gene coexpression, and transcription factor relationships – have been employed to infer functional associations that lend themselves to analysis as networks, in which each gene or protein is represented as a node and an interaction as an edge. Furthermore, network-based analyses can be used to identify biomarkers [6], to predict tumor progression [7], or to reveal the molecular alterations underlying disease [8].

However, our current knowledge of biological networks is far from complete. The coverage of current interactome databases is estimated to be less than 10% of the total number of interactions [9]. Thus, when interpolating the connections between driver genes, network-based analyses that rely solely upon confirmed interactions may lack essential connections. As one goal of our research is to predict and analyze the functional paths between driver genes, a critical step was to develop a predictive framework to infer and evaluate novel connections between genes. The framework proposed here (modeled on Pathfinder [10]) infers missing edges using predictions from protein family relationships and filters these paths based on known association rules. On the other hand, since a cancer gene participates in multiple signaling pathways, there may be dozens – if not, hundreds – of paths by which two proteins functionally interact. Thus, a computational approach is required to limit the network space to the specific biological context of interest. To extract functionally relevant subnetworks, the framework detects highly probable signaling pathways based on gene-gene mRNA coexpression and Gene Ontology [11] association rules mined from published pathways.

We used the computational method to elucidate the connections between a well-known driver gene of intestinal cancer, *Apc* (*adenomatous polyposis coli*), to another gene also involved in cancer, *Cdkn1a* (previously known as *p21*). Though *Cdkn1a* was not found to be mutated in populations of human colorectal cancers studied to date [2], its expression level correlates with neoplastic progression and has a prognostic value greater than that of *Trp53* [12]. Further supporting its importance in neoplasia, the double mutant mouse, *Apc^1638N+/−^ Cdkn1a^−/−^*, exhibits a synergistic increase in its tumor burden [13]. After predicting the network linking *Apc* and *Cdkn1a*, we evaluated the relevance of these predictions by manipulating the underlying system: generating *in vivo* network perturbations in two mouse models, followed by systems-level ‘omic measurements from the small intestinal epithelium. The ‘omic measurements – both proteomic and genomic – of the perturbed system were used for the statistical testing of the predicted network, thus introducing the concept of evaluating *in silico* predictions against context-specific biological data.

## Materials and Methods

### Network Analysis Framework

**Figure 1:**
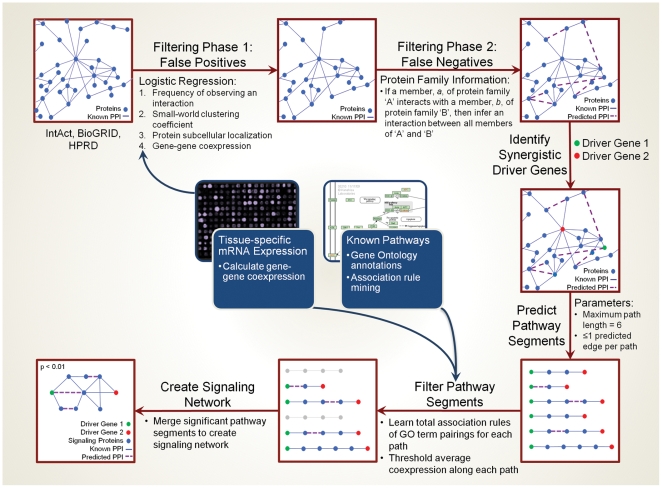
Framework for prediction of driver gene networks. The process begins with a two-step filtering process to account for false positives and false negatives in interaction databases. After selecting the driver genes of interest, pathways are predicted and then pruned using both GO term association rules and gene-gene coexpression values. Finally, the significant pathway segments are merged to arrive at a network connecting the two driver genes. The framework incorporates tissue-specific mRNA coexpression at two levels: in the pairwise filtering of false positives; and in the filtering of paths by average coexpression. The logistic regression model is trained on gold-standard interactome databases (see Methods S1 for additional details).

The network analysis framework (illustrated in Figure 1, and explained in the Methods S1) employs the PathFinder architecture outlined previously [10]. The raw network of publicly available physical interactions is first pruned of false positives using a logistic regression model that incorporates (i) the number of times a PPI is observed, (ii) the Pearson correlation of expression measurements for the corresponding genes, (iii) the proteins' small world clustering coefficient, and (iv) the protein subcellular localization data of interacting partners. Positive (1000 PPIs from the MIPS[14] database of interactions) and negative training data sets (1000 randomly selected PPIs that are not in MIPS) are used in 1000 cross-validation trials to acquire the parameters that maximize the likelihood of a true interaction.

False negative interactions are inferred using sequence homology relationships. It was observed that proteins with similar sequences share similar interaction partners in the same organism [15], and, thus, proteins from the same family are also likely to have similar interaction patterns. The Pfam database, utilizing multiple sequence alignments and hidden Markov models (HMMs), uses sequence similarity to formulate protein family classifications [16] and serves as a useful tool for exploiting these relationships. Hence, we inferred an interaction edge if (i) two proteins do not interact with each other in the PPI network, and (ii) there exists at least one interaction between the families of these two proteins.

To identify those paths relevant to our model system of interest, coexpression data based on microarray experiments from the *Apc^Min/+^* mouse small intestinal epithelium were obtained from the Gene Expression Omnibus (series GSE422 [17]); this study used laser-capture microdissection to sample the crypts of adenomas, carcinomas, and normal epithelium. In our implementation, we used Pfam release 23.0 [16] and the Gene Ontology release in August 2008 [11]. The search algorithm was extended to find pathways up to 6 nodes in length, and the threshold for the average coexpression of pathways was 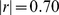.

### Mouse Intestinal Epithelium Isolation

All animals were handled in strict accordance with good animal practice as defined by the relevant national and/or local animal welfare bodies, and all animal work was approved by the Institutional Animal Care and Use Committee (IACUC) of Albert Einstein College of Medicine (permit number 20070805). *Apc^1638N+/−^* and *Cdkn1a^−/−^* C57BL6/J mice were generated as described previously [13] and tissue samples were harvested using the method outlined by Weiser et al., resulting in crypt and villus populations of cells from the small intestine of *Apc^1638N+/−^*, *Cdkn1a^−/−^*, and wild-type mice [18].

### 2D Differential In Gel Electrophoresis

2D Differential In Gel Electrophoresis (2D-DIGE) was performed as previously described [19]. Differentially expressed proteins from crypt and villus fractions were identified in the mutant mice (*Apc^1638N+/−^* and *Cdkn1a^−/−^*) relative to the respective fractions from wild-type mice (4 replicates each). Univariate t-tests (unequal variances and equal sample sizes) and multivariate linear regression (coded in the R package LIMMA [20]) were performed. Gel spots were selected for LC-MS/MS identification based on these two t-statistics at the 0.05 level of significance.

Gel spots were excised, trypsin digested, and the peptides were subsequently analyzed by tandem LC-MS/MS on a LC Packings/Dionex Ultimate 3000 HPLC-Orbitrap XL (Finnigan, San Jose, CA) system [19]. For interpretation of the MS/MS spectra, the MASCOT software package was used to search the SwissProt database; a null database of reversed peptide sequences was searched simultaneously to account for false positives. Identified proteins are listed in Table S1. Mascot DAT files have been made publicly available through the Proteomics Identifications Database [21], accession number 10638.

### Gene Expression Profiling

Microarray studies for crypt and villus populations from *Apc^1638N+/^* ^−^, *Cdkn1a^−/−^*, and wild-type mice (4 replicates each) were conducted on Affymetrix Mouse Genome 2.0 chips according to published procedures [22]. All data is MIAME compliant and the raw data have been made publicly available through the MIAME compliant database, the Gene Expression Omnibus [23], accession number GSE19338.

### Network mRNA Analysis

Raw .CEL files were processed in MATLAB using the Robust Multiarray Averaging procedure [24]. To deal with multiple probes capturing different aspects of a gene product's behavior, we used all probes to represent a gene. Thus, in the following analysis, each *Apc-Cdkn1a* network node, *i*, was represented by *k_i_* probes on the array, resulting in a matrix of size *q*×*n*, where 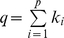 and 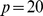. To determine whether the *Apc-Cdkn1a* network nodes were collectively differentially expressed in a tissue compartment (crypts or villi), we extended Hotelling's *T^2^* statistic – a classical approach useful for testing gene groups [25] – to incorporate multiple experiments, as follows:
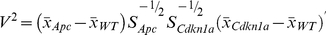
Where 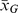 is the vector of mean mRNA intensity for all the *q* probes for a genetic background, *G*, where 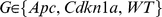 (*Apc* indicating *Apc^1638N+/−^*; *Cdkn1a* indicating *Cdkn1a^−/−^*; and *WT* indicating wild-type C57BL6/J). *S* is the absolute value of the unbiased pooled sample covariance matrix for each mutant:
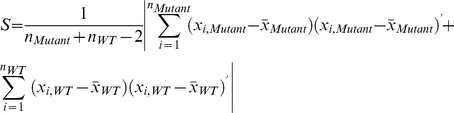
Where *Mutant* can refer to either *Apc^1638N+/−^* or *Cdkn1a^−/−^*, and the absolute value in *S* is used to avoid imaginary components when taking the inverse root of *S* in 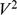. It should be noted that probes corresponding to *Apc* and *Cdkn1a* themselves were excluded, as these are expected to have extremely low intensity values (in the respective mutants) that would skew the perceived aggregate network effect. In 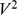, the difference of means, 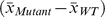, for each mutant may be positive or negative for a probe *i*, so, unlike *T^2^*, *V^2^* can be either positive or negative.

Given that 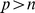, sample covariance estimates are not positive definite, and hence, the inverse is singular. To circumvent this issue, we set all covariances to zero for initial calculation of *V^2^* and then calculate the significance of *V^2^* using a permutation test (i.e. stochastically generating new “*mutant*” and “*wild-type*” phenotype labels), thus preserving the underlying covariance structure in the null distribution. Setting the off-diagonal elements of *S* to zero simplifies *V^2^* to:
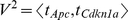
Thus, *V^2^* is simply the sum of the product of scaled t-statistics calculated for each probe, in each of the two experimental perturbations. As the number of samples was small (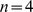 for mutant and wild-type, each), random 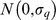 noise was added to each permutated matrix to obtain an interpolated and smoothed empirical null distribution; the standard deviation, 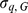, of the noise for each probe, *q*, in the genetic background, *G*, was estimated by the sample standard deviation of each probe. 10000 such permutations were calculated to obtain the null distributions, which –as expected – resemble F-distributions (see Figure S1). Since *Apc* and *Cdkn1a* are both tumor suppressors and hypothesized to affect our network of interest in a similar fashion, we expect the t-statistics to vary in the same direction if the null hypothesis (of no joint effect) is to be rejected. Hence, we compute the *p*-value of *V^2^* as the number of null observations greater than our observed value of *V^2^*. Calculating the *p*-value for the negative tail of the distribution would be useful if the perturbations were expected to have opposite molecular effects (e.g. *Apc^+/−^* paired with a *Stat3^+/−^* hypomorph).

While we present an analysis for a 2-node perturbation of a network, this analysis is extensible to *k* experimental perturbations by computing pairwise *V^2^* statistics, resulting in a matrix:
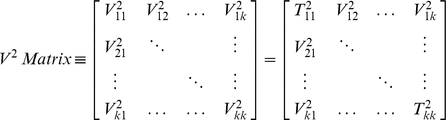
Where 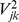 represents the 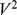 statistic between perturbations *j* and *k*; as shown, the diagonal reduces to a scaled version of Hotelling's *T^2^* statistic for each experiment. As the statistics are each of a different scale, they cannot be compared directly, and, therefore, the significance of each matrix element should be calculated (as above) via a permutation test. Then, for the matrix of *p*-values, the diagonal elements provide information about the significance of individual experiments, while the off-diagonal values provide information about pairwise experimental significance. The total experimental support for network perturbations can then be calculated by aggregating off-diagonal *p*-values, e.g. by Fisher's method [26]. We recommend this approach for dealing with 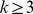 perturbations; for 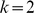 perturbations, as in our case, the *p*-values can be interpreted directly.

### Analysis of Proteomic Targets

To assess the importance of physical proximity, the topological distance between *Apc*-*Cdkn1a* network nodes and the respective proteomic targets was calculated. Physical PPI networks were assembled from BioGRID [27], the Human Protein Reference Database (HPRD) [28], and IntAct [29]. Each network node was tested independently for the number of 2-hop paths connecting it to a set of *n* experimentally measured proteins, expressed as follows:
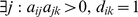


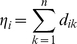
Where 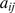 is the entry at row *i* and column *j* in the adjacency matrix, *A*, of the PPI network; *i* is a protein in the *Apc-Cdkn1a* network; *j* is an intermediate protein; and *k* is an experimentally measured protein. In this case, the experimental proteins were the proteomic targets from either *Apc^1638N+/−^* or *Cdkn1a^−/−^* mice. If there is at least one intermediate protein, *j*, for which a two-hop path exists between nodes *i* and *k*, then the 2-hop distance, 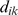, is 1; the total connectivity, 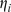, of protein *i* to the set of 2D-DIGE targets is simply the sum of the 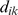. Significance was calculated against an empirical null formulated from 10000 randomly generated sets of proteins also of size *n*.

To assess patterns of coregulation, mRNA coexpression values (Spearman's correlation coefficient) were calculated from the corresponding set of normalized microarray experiments, spanning wild-type, *Apc^1638N+/−^*, and *Cdkn1a^−/−^* crypts and villi; the probe with maximum intensity was used as the representative for a gene. To test the significance of mRNA-level correlations, a modified Kuiper's test statistic, *K*, was calculated between the group correlations (i.e. all probes on the array) and sample correlations (i.e. set of 2D-DIGE targets) for each node in the network independently; it is calculated as the sum of the maximal and minimal deviations of the sample, 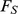, and control (i.e. entire array), *F*, cumulative distribution functions [30]:
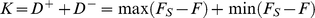
As per the suggestions of Subramanian et al. [31], the Kuiper's statistic, *K*, was modified to improve its ability to detect bimodal shifts in location of the sample distribution (as one would expect coexpressed groups of proteins to show both positive and negative correlations):
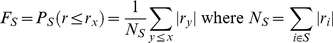
Where *S* is the set of proteins being tested (either the *Apc^1638N+/−^* or *Cdkn1a^−/−^* 2D-DIGE targets); *r* is the ordered vector of correlation coefficients between the respective 2D-DIGE targets and a single network node; and 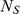 normalizes 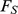 to have sum 1. Significance testing was performed using a normal approximation of the empirical null: the empirical null was assembled from the modified *K* calculated for 500 randomly selected protein sets, each of size 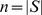, and maximum likelihood estimation was used to fit a normal distribution. For exploring and illustrating the connections of significant (*α* = 0.05) network nodes, we examine the subset of correlations, *r_y_*, where 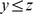 such that 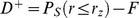 and 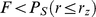; and the subset of correlations, *r_p_*, where 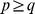 such that 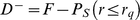 and 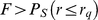 (analogous to the “leading edge” subset of GSEA [31]). To identify differentially expressed nodes, we chose those nodes where the t-statistic (unequal variance) of the maximum intensity probe was such that 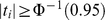 in either the crypt or the villus compartment, where 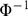 is the normal inverse cumulative distribution function.

Testing each node in the *Apc-Cdkn1a* network independently resulted in a *p*-value for each of the 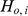 null hypotheses, where 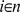, and each hypothesis, 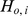, assumes that there is no relationship (physically-based or coexpression-based) between the *Apc-Cdkn1a* network node, *i*, and the 2D-DIGE targets. To test the group null hypothesis that all 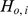 are simultaneously true, *p*-values were aggregated into a statistic, *τ*, suggested by Fisher; significance was assessed against a 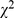 distribution with 2*n* degrees of freedom [26] (see also Methods S1). The mutated node (*Apc* in *Apc^1638N+/−^* or *Cdkn1a* in *Cdkn1a^−/−^*) was excluded from the respective analyses, as their extreme expression patterns skew the group-wise results.

## Results

### Driver Gene Network Predictions

The double mutant *Apc^1638N+/−^ Cdkn1a^−/−^* mouse was previously shown to exhibit a synergistic increase in its tumor burden when compared with the single mutants [13]. To identify the potential connections between *Apc* and *Cdkn1a*, we constructed a predictive framework that, first, learns the annotation patterns characteristic of known signaling pathways (e.g. those found in KEGG [32] and others) and, then, couples these patterns with tissue specific coexpression data to extract the most likely chains of interacting proteins involved in *Apc-Cdkn1a* signaling (illustrated in Figure 1). To identify only high-confidence pathways, a two-phase filtering process was first applied to the global PPI network. In the first phase, edges – compiled from mammalian interactions in BioGRID [27] and HPRD [28] – were pruned from the network if they did not resemble likely interactions (as defined by a logistic regression model), with the goal of reducing false positives among the reported interactions. To account for false negatives (Phase 2), interactions were added to the network by inferring relationships that are precedented in model organisms based on protein family relationships. After applying these measures to generate a synthetic network, we searched for likely connections between *Apc* and *Cdkn1a* using both gene coexpression data and Gene Ontology association rules.

**Figure 2:**
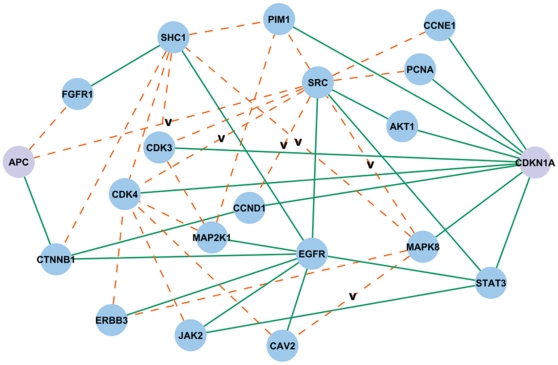
The *Apc*-*Cdkn1a* network. Solid edges represent previously known interactions; dashed edges represent predicted interactions; and edges marked with a “**v**” represent predicted interactions that have been validated recently in the published literature.

To emphasize nodes and edges relevant to our biological system, we introduced a tissue-specific bias in our search for *Apc*-*Cdkn1a* connections by using gene expression data from the intestinal epithelium of *Apc^Min/+^* mice. From these data, we calculated the mRNA-level coexpression value for individual edges via the gene-gene Pearson correlation coefficient. Next, all paths in the synthetic network linking the gene products of *Apc* and *Cdkn1a* were queried, and the predicted paths were filtered based on (i) the support of association rules for GO annotations and (ii) the average coexpression along a path; the result (at a significance level of *α* = 0.01) is shown in Figure 2. The *Apc*-*Cdkn1a* network includes a number of previously known interactions (solid lines), as well as predicted interactions (dashed lines) based on: (i) protein family relationships, (ii) strength of GO association rules, and (iii) microarray coexpression along the specific path connecting *Apc* to *Cdkn1a*. As genetic interactions were included in the original interaction databases, the predicted network includes both physical and functional relationships.

At a systems-level, the proposed *Apc-Cdkn1a* network bears the statistically unlikely property of being saturated with oncogenes: 8 of the 20 proteins are annotated as oncogenes in OMIM (*p*-value<5×10^−10^ by Fisher's exact test, see Methods S1), and many of the remaining genes have been experimentally shown to act as oncogenes (e.g. *Erbb3* [33], [34], *Shc1* [35], *Map2k1* [36]). Although the *Apc*-*Cdkn1a* network contains many well-studied proteins, the node degree (i.e. number of interactions) within the subnetwork does not strictly correlate with the node degree in the unfiltered interaction database (Pearson's correlation = 0.51). For instance, while AKT1 has many known interactions, its commonly studied biological partners – namely, GSK3B and PTEN (both of which are associated with *Apc* [3] and *Cdkn1a* [37] signaling) – do not appear in the network. Other known interactions, such as that between SHC1 and SRC [38], are also absent from the network. Since our algorithm predicts connections biased by the biology of the system under study (through the use of gene expression data from *Apc^Min/+^* mouse intestinal tissue), a particular protein or edge may not appear in the network if the pathway (i.e. chain of proteins) on which it resides does not meet the gene coexpression and/or GO association rule thresholds.

Conversely, the *Apc*-*Cdkn1a* network includes novel associations: those not contained within the source databases (dashed edges in Figure 2). Several of these interactions have recently been validated in focused studies (see Table 1), providing confidence that the framework is useful. In addition, the *Apc*-*Cdkn1a* network also suggests that certain interactions previously associated with other cancer models – such as the SRC-CCND1 functional association found in prostate cancer [39], or the phosphorylation of CDK4 by SRC in a cell line [40] – are relevant in this model of colon cancer.

**Table 1:** Published evidence validating interactions predicted in the *Apc-Cdkn1a* network.

Protein A	Protein B	Interaction Type	System	Description
**MAPK8**	CAV2^a^	Functional	Human, fetal fibroblasts;Human, lung tissue	CAV1 forms hetero-oligomers with CAV2, and CAV1 inhibits TGF-beta or IL-6 induced phosphorylation of Mapk8 in fibroblasts [51]
		Functional	Human, gingival fibroblasts	siRNA knockdown of CAV1suppressed MAPK8 phosphorylation [52]
**SRC**	APC	Functional	Mouse, colon epithelial cell line	Stable expression of SRC resulted in increased proliferation of *Apc^Min/+^* cells versus *Apc^+/+^* cells [53]
**SRC**	CCND1	Functional	Human, breast cancer cell line	SRC transfection leads to CCND1-CDK4-p27 complex formation [54]
		Functional	Human, prostate cancer cell line	SRC inhibition resulted in decreased binding of β-catenin to the promoters of G1 phase cell cycle regulators cyclin D1 and c-Myc [39]
		Functional	Mouse, renal cell line	siRNA knockdown of SRC decreased CCND1 expression [55]
**SRC**	CDK4	Phosphorylation	Human, colon cancer cell line	SRC phosphorylates CDK4 [40]
**SRC**	PCNA	Functional	Human, ovarian cancer xenograft	Administration of a small molecule inhibitor of SRC results in decreased staining for PCNA in mouse carrying the xenograft [56]
**CDK4**	CAV2^a^	Functional	Mouse, ES cells	Expression of CDK4 decreased upon knock-down of Caveolin-1 [57]

a Though CAV2 was discovered in the subnetwork, CAV1 and CAV2 are located adjacent to each other on chromosome 7 and express co-localizing proteins that form a stable complex.

### Single Node Perturbations: mRNA Profiling

As the *Apc-Cdkn1a* network represents the intersection of signaling pathways emanating from *Apc* and from *Cdkn1a*, we expect to observe functional changes in network-associated proteins in response to perturbations at either *Apc* or *Cdkn1a*. Single-node perturbations were developed in mouse models with mutations in either *Apc* (namely, *Apc^1638N+/−^*) or *Cdkn1a* (*Cdkn1a^−/−^*). While the *Apc*-*Cdkn1a* network was generated using tumor-specific *Apc^Min/+^* data – a model harboring a number of background genetic lesions [41] – the intestinal tissue obtained from the *Apc^1638N+/−^* and *Cdkn1a^−/−^* mice at 3 months of age is relatively polyp free, thus allowing us to gauge the effect of a single genetic perturbation on the pre-neoplastic epithelium. Although this removes potential bias that is introduced by subsequent mutations of neoplastic tissue, this approach may also attenuate the flow of information between the two genes.

**Figure 3:**
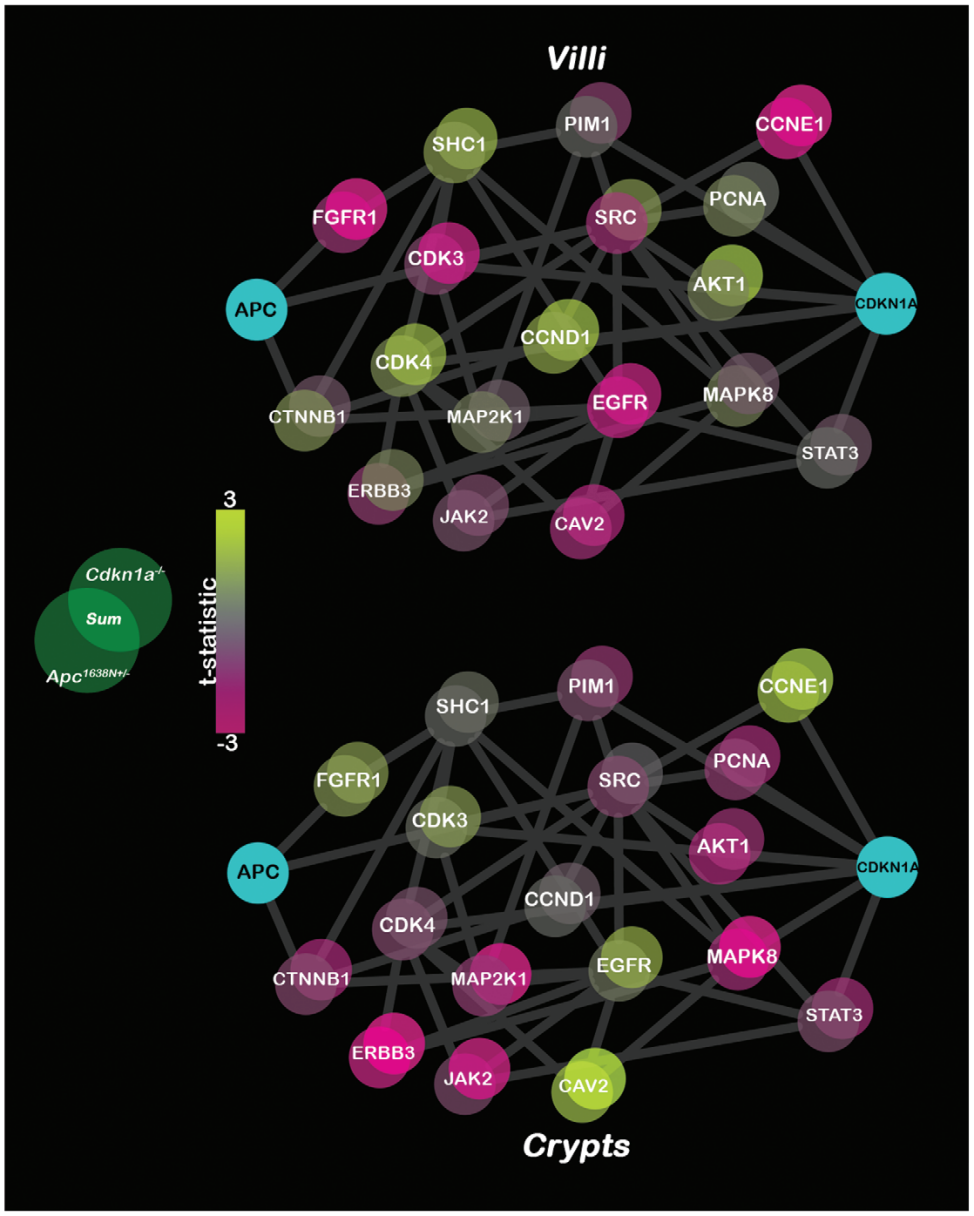
Differential expression of the *Apc*-*Cdkn1a* network in villi (top) and crypts (bottom) for the *Apc^1638N+/−^* and *Cdkn1a^−/−^* mouse models. Each network gene is represented by two overlapping bubbles colored according to the t-statistics (unequal variance) in the two mutants: the lower left bubble of a gene corresponds to the t-statistic for *Apc^1638N+/−^*, and the upper left bubble to the t-statistic for *Cdkn1a^−/−^*. The intersection of the two bubbles corresponds to the sum of the t-statistics, illustrating how the significance of small effects can be strengthened when considered jointly. Nodes downregulated in the mutant are colored pink, those upregulated in the mutant are yellow, and neutral t-statistics are grey. While *V^2^* is calculated using all probes for each gene, we use only the probe with maximum intensity to calculate the t-statistics for visualization.

Since we are using the two perturbations to determine how well the *Apc-Cdkn1a* network can capture biological phenomena, we introduced a multivariate statistic, *V^2^* to test if differences in mean mRNA abundance exist jointly between the *Apc^1638N+/−^* and *Cdkn1a^−/−^* models. By using *V^2^*, as illustrated in Figure 3, genes with mild differential expression in the two individual mutants can contribute to the overall support of the network, as *V^2^* rewards those genes where each of the two independent t-statistics are both greater than 1. Statistical significance of *V^2^* was tested against a permutation null, and, as our perturbations involved two tumor suppressors expected to have molecular effects in the same direction, we used the positive tail of the distribution. Knowing that many molecules “switch” expression (i.e. high to low, or vice versa) in the transition from crypts to villi [19], the microarray datasets for these two biological compartments were tested separately. We found that the *Apc-Cdkn1a* network was strongly supported (*p*-value = 0.002) by the joint mRNA differential expression in the two mutants' crypt compartment. Network coherence was weaker (*p*-value = 0.060) in the villus compartment, and the network as a whole was not differentially expressed in the villi of either mutant, noted in the two *V^2^* matrices' *p*-values:
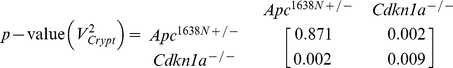


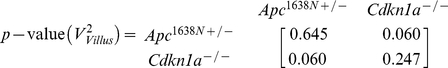
Where, as mentioned, the diagonal elements indicate the significance of differential expression *within* a mutant (as per Hotelling's *T^2^*), and the off-diagonal elements indicate significance of joint differential expression *across* mutants (as per *V^2^*). In the crypts, the network was differentially expressed in *Cdkn1a^−/−^* (*p*-value = 0.009), but not in *Apc^1638N+/−^* (*p*-value = 0.871), and, yet, was jointly supported by differential expression across both mouse models (*p*-value = 0.002). This illustrates that small mRNA-level changes that are shared between multiple perturbations – on a gene-by-gene basis – provide joint support for the network hypothesis, while any individual perturbation may fail to demonstrate the claim.

To illustrate how the joint consideration of gene-wise behavior operates, each network node has color-coded bubbles for the t-statistics of both *Apc^1638N+/−^* and *Cdkn1a^−/−^* in Figure 3; the sum is shown at the intersection of each gene's bubbles. Though *V^2^* employs products of t-statistics, the sum is better suited for visually demonstrating the principle that small mRNA effects can have a significant impact when considered together. We observe that several nodes that are differentially expressed in the crypts – ERBB3, JAK2, MAPK8, et al. – are no longer differentially expressed in the villus. In addition, some genes – e.g. CCNE1, CAV2, FGFR1, EGFR – switch their direction of expression between the crypts and villi.

### Single Node Perturbations: Proteomic Profiling

**Figure 4:**
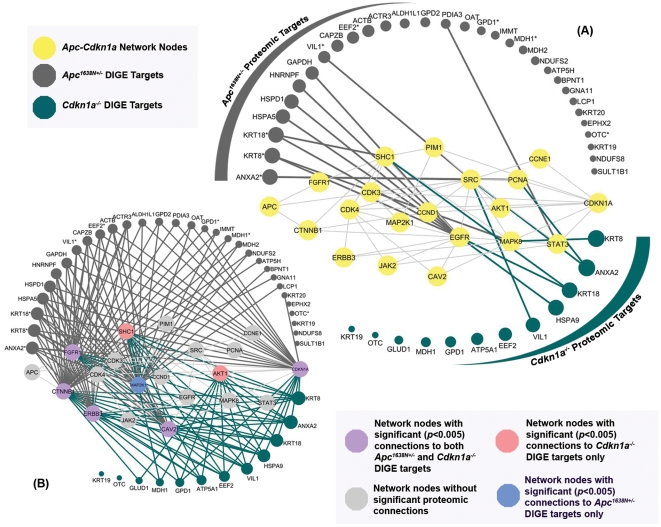
Physical connections between the 2D-DIGE targets and the *Apc-Cdkn1a* network. (A) Direct physical linkages between 2D-DIGE targets and network nodes. (B) Indirect (2-hop) physical linkages between 2D-DIGE targets and network nodes. Node size corresponds to the number of 2-hop interactions it possesses. *Apc^1638N+/−^* 2D-DIGE targets marked with a “*” were also found in the *Cdkn1a^−/−^* intestinal epithelium.

The 2D-DIGE analysis reported 12 proteins differentially expressed for the *Cdkn1a^−/−^* intestinal epithelium (crypts and villi combined) versus wild type, and 31 proteins differentially expressed in the epithelium of the *Apc^1638N+/−^* mice versus wild type (Table S1). To test our network-based hypothesis, we first assumed that the set of regulatory molecules in the *Apc-Cdkn1a* network are independent. Then, the one and two-hop physical interactions were assessed for each network node (see Figure 4). While directly interacting neighbors (one hop) are typically useful in mapping signaling pathways, they did not associate much of the proteomic data with the network. Also, the few direct connections were not statistically significant; EGFR, for example, tends to have many interactions, and, thus, EGFR's direct connections to the 2D-DIGE targets were not more likely than expected by chance. However, analysis of indirect interactions (two hops) from network nodes captured the relationship to the majority of the 2D-DIGE targets (individual node's *p*-value <0.005), as illustrated in Figure 4B. Considering the network as a whole and aggregating the *p*-values (aggregate statistic, *τ*), the network was significantly (*p*-value of *τ*<1×10^−15^) physically associated with either the *Apc^1638N+/−^* or *Cdkn1a^−/−^* 2D-DIGE targets, suggesting that the proteome-level effects are at most 4-hops away from the causative mutations.

**Figure 5:**
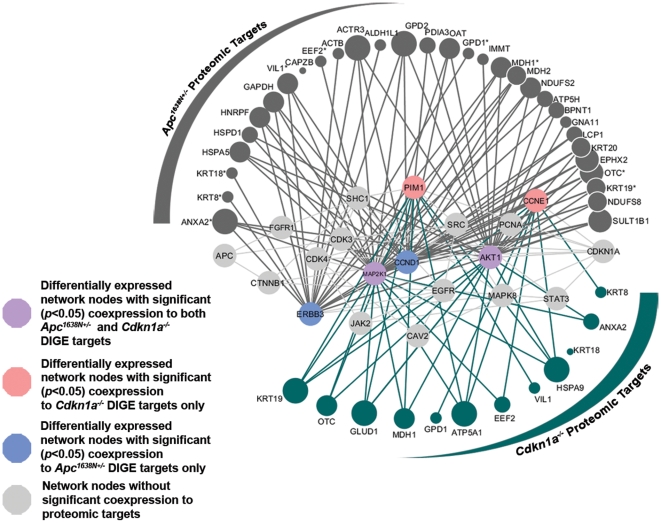
Coexpression between the 2D-DIGE targets and the differentially expressed *Apc-Cdkn1a* network nodes. To examine the network-based hypothesis, each network node was tested independently for significant correlation with the *Apc^1638N+/−^* or *Cdkn1a^−/−^* 2D-DIGE targets using a modified Kuiper's test statistic. The 2D-DIGE targets are ordered by the amount of second-degree physical interaction, per Figure 4; node size is proportional to the number of coexpression interactions with differentially expressed signaling proteins. *Apc^1638N+/−^* 2D-DIGE targets marked with a “*” were also found in the *Cdkn1a^−/−^* intestinal epithelium.

Based on the physical proximity of the 2D-DIGE targets to the *Apc-Cdkn1a* network, we hypothesized that these network proteins might be controlling the expression of the 2D-DIGE targets. To examine this relationship further, we studied the pattern of mRNA-level coexpression between network nodes and the 2D-DIGE targets. As before, the network nodes were assumed to be independent, and the pattern of coexpression was assessed for each node individually using a modified Kuiper's test statistic, *K*; nodes identified as (i) being differentially expressed (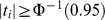) in either crypts or villi and (ii) having significant (*α* = 0.05) coexpression with the 2D-DIGE targets are highlighted in Figure 5. Fifteen nodes in the *Apc-Cdkn1a* network had significant mRNA-level correlations to the *Apc^1638N+/−^* 2D-DIGE targets, and four of these were also differentially expressed. On the other hand, eight nodes had significant correlations to the *Cdkn1a^−/−^* 2D-DIGE targets, and four of these were individually differentially expressed. Considering coexpression relationships from the*Apc-Cdkn1a* network as a whole, the *p*-value of *τ* for coexpression between *Apc-Cdkn1a* network nodes and *Apc^1638N+/−^* 2D-DIGE targets was strongly significant (all nodes excluding *Apc* and *Cdkn1a*, *p*-value<1×10^−20^; differentially expressed nodes, *p*-value = 1.4×10^−5^). Given the magnitude of these group-wise statistics, however, the evidence for *Apc-Cdkn1a* network coexpression with the *Cdkn1a^−/−^* 2D-DIGE targets was not as well-supported (all nodes, *p*-value of *τ* = 3.1×10^−8^; differentially expressed nodes, *p*-value of *τ* = 1.6×10^−3^). Given that *τ* can be influenced by a few small *p*-values, we also calculated the probability of observing *k p*-values less than *α* = 0.05, which, as a binomial distribution, is more sensitive to larger *p*-values. This also indicated that the *Cdkn1a^−/−^* 2D-DIGE targets were least supported by coexpression with the *Apc-Cdkn1a* network, with the *p*-values separated by two orders of magnitude again (*p*-value for coexpression of *Cdkn1a^−/−^* targets with differentially expressed network nodes was 3.3×10^−5^; *p*-value for coexpression of *Apc^1638N+/−^* targets was 3.1×10^−7^).

Finally, knowing that the use of a single probe per gene is often misleading, the above calculations for proteomic coexpression were also performed using all microarray probes for the network nodes and the proteomic targets. By this approach, the network as a whole was strongly coexpressed in either mutant (for differentially expressed probes, excluding those belonging to *Apc* or *Cdkn1a*, *p*-value of *τ*<1×10^−20^). However, for ease of interpretability and visualization, we discuss the results of the analysis using only the maximum intensity probe per gene.

## Discussion

As underscored by Wood et al. [2], colorectal cancer is the product of mutations in multiple genes operating simultaneously. Though tumors differ at the genetic level, their phenotypes intersect at histopathologic levels and, in our view, at the molecular level, as well, implying that the connections between unique sets of mutations may often merge at downstream signalling hubs. Thus, the reduction of genetic heterogeneity into clinically meaningful biological networks could have an impact in the context of personalized medicine. As a step towards this goal, we developed a computational framework capable of predicting functional connections between genes mutated in cancer, and we applied our methodology to define a network between *Apc* and *Cdkn1a*.

Networks, as abstractions of underlying molecular phenomena, offer the hope of distilling system-level structure from biological complexity. Given the many degrees of freedom in the underlying datasets (PPIs, microarrays, et al.), however, numerous network structures are possible for a particular biological context, and candidate networks are often evaluated solely based on topological significance (e.g. the G-score of MetaCore [42]). Yet, the value of a network may not be reflected topologically; for example, a highly-connected hub may not be highly active in a particular model system. If a network model truly reflects the underlying biology, then – from an engineering standpoint – perturbations of the underlying system should be manifest in and around the predicted network. Specifically, since a network connecting two cancer genes represents cross-connections between signaling pathways, one would expect that perturbations along a pathway would result in altered regulation of network-associated nodes.

A systems-based approach required to biologically evaluate network coherence, however, is not immediately amenable to the tools of classical molecular biology, which are designed to target single molecules or, at most, a few at a time. As an alternative, we outline the use of *in vivo* single node perturbations – by way of mouse models with targeted inactivation of specific loci – followed by gene and protein expression analysis to gauge systems-level effects. For further investigation, numerous mouse models for cancer biology are already available, and these resources can be productively mined to expand our understanding of cancer gene networks. Though we have demonstrated the value of biologically testing network predictions using 2D-DIGE and microarray data, many other types of screening tools could also be employed to test the functional coherence of predicted networks.

Due to the differences in coverage of proteomic and gene expression data, different approaches were required to probe the potential functional coherence of the network: *V^2^* – a multivariate statistic – was used to gauge the effect of single node perturbations on mRNA-levels of the *Apc*-*Cdkn1a* network, while, due to reduced coverage, relational maps – physical and coexpression – were required to assess the effect of driver gene mutations on the proteome. We found that the *Apc-Cdkn1a* network was supported by the joint differential expression of mRNA in two different network perturbations, with stronger differential expression being observed in the crypts (Figure 3). The *V^2^* statistic is presented in a framework that is extendable to multiple network perturbations – a feature that proves necessary in evaluating the biological coherence of networks, as small mRNA-level effects of an individual perturbation may fail to lend adequate support for a predicted network; coupling multiple perturbations together via the *V^2^* matrix allows the integrity of the network to be assessed via a biologically multidimensional approach. It should be noted that, in testing the mRNA-level support for an individual network, a “self-contained” hypothesis is necessary, embodied by null distributions – such as the permutation null used here – modeling the population from which the samples (mice) were drawn; gene randomization methods, on the other hand, compare network expression patterns to stochastically chosen gene groups, which are bound to have a different and/or reduced covariance structure (especially without incorporating network structure to generate the null gene sets), leading to overinflated significance values [43].

After applying *V^2^* to the mRNA data, we found that the network was better able to capture joint differential expression in the crypts than in the villi, suggesting that oncogenic transformations are initiated in the crypts by the network genes and then transduced to downstream targets in the villi. This is reflected in Figure 3, where the t-statistic of individual nodes is colored for both mouse models, and more nodes are seen to be brightly colored (i.e. highly differentially expressed) in the crypts. Interestingly, a large contingent of oncogenes – ERBB3, JAK2, MAP2K1, MAPK8 – are clearly downregulated in the crypts, while their expression levels diminish considerably in the villi, indicating that these genes turn “off” during the crypt-to-villus transition – a well-known feature of this biological compartment [19]. Though the downregulation of oncogenes may appear counterintuitive, it is to be expected in these particular mouse models, where the tumorigenic phenotype is mild and the tissue has been harvested in the pre-neoplastic regime. Before the onset of tumors, the downregulation of oncogenes represents a homeostatic reflex of the tissue to the genetic perturbations, i.e. protective downregulation of oncogenes to compensate for the loss of *Cdkn1a* or *Apc*. In addition to genes turning “off” in the crypt-to-villus transition, several genes appear to switch their pattern of expression entirely. In particular, CDK3, CAV2, EGFR, and FGFR1 exhibit this behavior, which suggests that they play two different roles in the two compartments.

For visualization, we show only the probe with maximum intensity across all samples for each gene. Given the extent of alternative splicing and array manufacturing variation, however, a single probe can be misleading. Hence, for calculation of statistical significance, we use all probes to model a single gene – a more robust approach that is amenable to matrix-based calculations (such as *V^2^*). We concede that, since each gene is represented by a different number of probes, genes with many probes contribute proportionally more weight to the final *V^2^* statistic. This is a useful feature, however, as we have more confidence about the true behavior of these well-probed genes – many of which are well-studied and important in cancer, such as *Egfr* (7 probes) and *Mapk8* (5 probes) – and, thus, they deserve greater weight than the highly variable, single-probe genes.

Proteomic data, however, requires different analytical considerations, as the protein levels of the network nodes may not be directly measured in a given proteomic experiment. To make inferences about our network-based hypothesis, we used two different mappings: one based on physical interactions, and another based on mRNA correlations. From Figure 4, it is clear that the proteins measured in the 2D-DIGE experiment are not merely a random sampling from the proteome. Rather, they are physically close to (i) the hypothesized network as a whole (*p*-value of *τ*<1×10^−15^) and (ii) several individual signaling molecules (individual *p*-values<0.005). Specifically, the 2D-DIGE targets from the *Apc^1638N+/−^* and *Cdkn1a^−/−^* experiments have significant physical proximity to CTNNB1, FGFR1, ERBB3, CAV2, and CDKN1A itself. The tight physical proximity of the predicted network nodes to experimentally measured targets suggested that the signaling molecules more proximal to the mutations may regulate the proteomic targets, and we used mRNA-level coexpression to examine this relationship further. While coexpression relationships abound between the *Apc^1638N+/−^* 2D-DIGE targets and the *Apc-Cdkn1a* network nodes, this is less significant for the *Cdkn1a^−/−^* targets (*p*-value of *τ* = 3.3×10^−3^). Taken together with the results of the mRNA analysis, this suggests that the hypothesized network nodes more effectively capture *Cdkn1a^−/−^* signaling at the mRNA level rather than at a proteomic level, whereas the opposite is true of *Apc* signaling.

While the network as a whole showed differences in the level of proteomic coexpression, two differentially expressed nodes – MAP2K1 and AKT1 – were significantly coexpressed with the measured proteome in both network perturbations (Figure 5). Interestingly, AKT1 was also found to be closely physically associated with the proteomic targets in *Cdkn1a^−/−^*, while MAP2K1 was physically associated with *Apc^1638N+/−^* 2D-DIGE targets (Figure 4). As we know that mRNA coexpression can provide evidence regarding the regulatory role of proteins [44], the mutual discovery of MAP2K1 and AKT1 in the two network perturbations – via both coexpression and physical connectivity to the perturbed proteome – suggests that these two proteins may serve as intersection points of *Apc* and *Cdkn1a* signaling. Also of interest is the observation that coexpression connections and physical connections tend to associate different subsets of the proteomic targets, as the more physically distant proteomic targets (e.g. SULT1B1, OTC, KRT19) are also the ones that tend to be coexpressed with multiple network nodes. Not only does this illustrate that physical and coexpression maps capture different dimensions of biological function, but it also illustrates the necessity of using both maps to provide complementary information in evaluating the molecular context of network hypotheses.

While the 2D-DIGE studies revealed many differentially expressed proteins, annexin A2 (ANXA2) was among the most highly ranked in its physical proximity to the hypothesized network. At the protein level, ANXA2 was upregulated in both mouse models of colon cancer (see Table S1). From studies of prostate cancer [45], ANXA2 upregulation is expected since *in vitro* experiments indicate that overexpression promotes a more invasive, proliferative cell phenotype. Though ANXA2 had high mRNA expression in one population of human colorectal tumors [46], it is downregulated in some populations of human prostate tumors [45] and colorectal cancer cell lines [47]. Since activation of either *Apc* or *Cdkn1a* signaling leads to upregulation of ANXA2 in our studies, activation of alternative or repressive pathways may lead to such unexpected downregulation of ANXA2 in some tumors.

In addition to ANXA2, the mutant mice exhibited other protein-level alterations that can potentially contribute to tumorigenesis. Elongation factor E2 (EEF2), for example, was found to be upregulated in both mouse models, and its tumorigenic potential in gastrointestinal [48] and breast [49] cancers is well known. Though drugs inhibiting the EEF2 pathway (via its kinase) exist [50], and molecular chemotherapy targeting ANXA2 can also be envisioned, our network-based hypothesis suggests these changes may be controlled by specific upstream signaling molecules that integrate the information from mutated genes. Thus, in patients where levels of ANXA2 or EEF2 are elevated, molecular therapy targeting the proposed upstream network targets – such as MAP2K1 or AKT1 – may be more effective.

In conclusion, we outline a novel method for identifying networks that connect signaling pathways associated with cancer driver genes. The first step towards statistically analyzing these novel subnetworks was pursued using single node perturbations of the system *in vivo*, followed by network interrogation via high-throughput -omics experiments. Together, the various lines of evidence – mRNA differential expression, 2D-DIGE-target physical proximity, and 2D-DIGE-target coexpression – strengthen the hypothesis that the *Apc*-*Cdkn1a* network helps to mediate both *Apc* and *Cdkn1a* signaling. Thus, we show that using ‘omic data to test a network-based hypothesis not only allows one to assess the biological validity of *in silico* predictions, it also allows one to prune the hypothesis to identify molecular targets (e.g. SRC and EGFR) that are likely to integrate the various signaling pathways perturbed in cancer.

## Supporting Information

Figure S1 Null distributions of *V^2^* in the crypts and villi.
(0.11 MB DOC)

Methods S1 Methods covering the statistical analysis of 2D-DIGE targets; the analysis of OMIM; and the construction of the filtered protein-protein interaction network.
(0.08 MB DOC)

Table S1 Lists of 2D-DIGE targets identified in the intestinal crypts and villi of the *Apc^1638N+/−^* and *Cdkn1a^−/−^* mice.
(0.12 MB DOC)
