# Supplementary material for: Prediction and Testing of Biological Networks Underlying Intestinal Cancer
Source: PLoS One. 2010 Sep 1;5(9):e12497. doi: 10.1371/journal.pone.0012497 (PMC2931697; doi:10.1371/journal.pone.0012497)
Supplement: Figure S1 — Null distributions of V2 in the crypts and villi. (0.11 MB DOC) [file pone.0012497.s001.doc]

**Supplementary Figure S1:**

**Null Distributions of *V^2^***


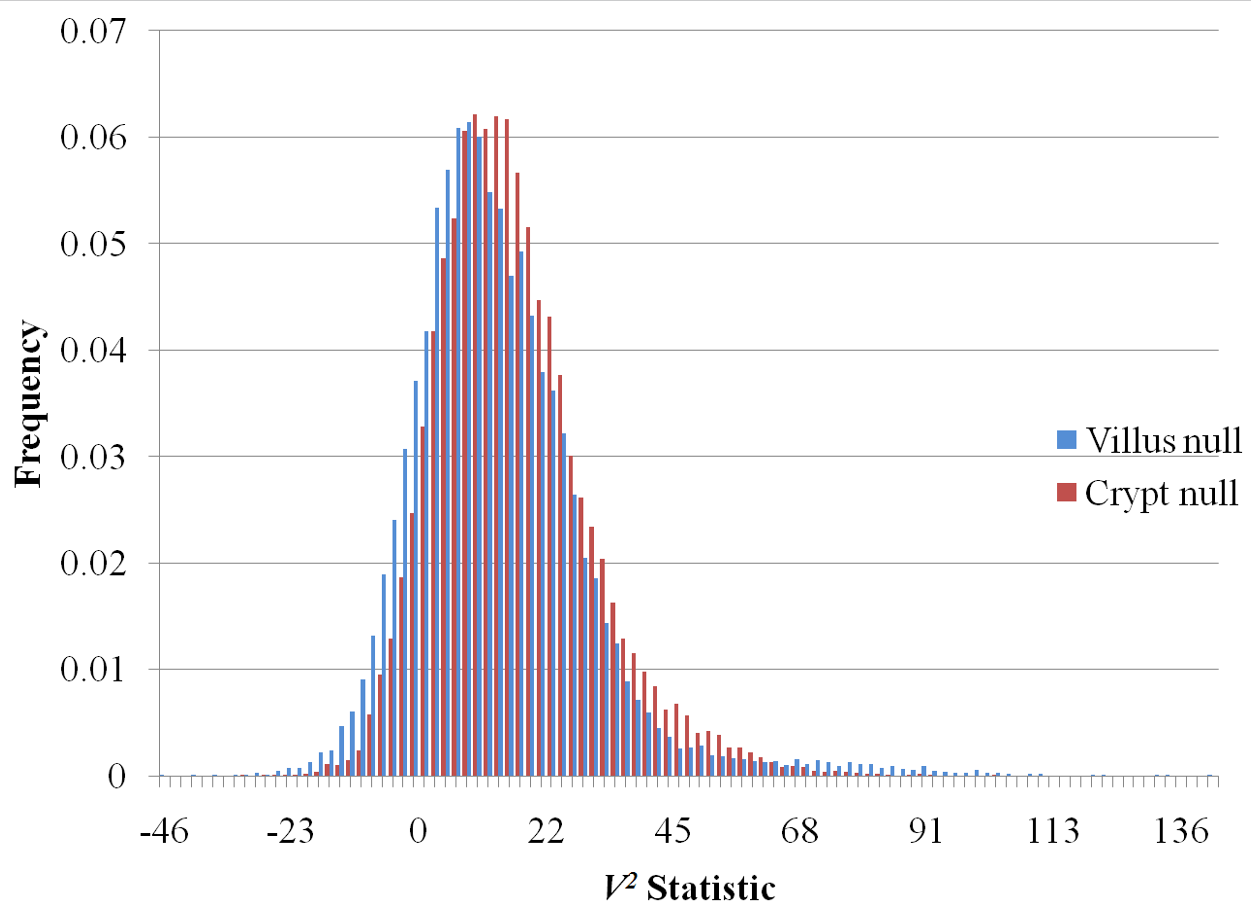


Figure S1. Empirical null distributions of the *V^2^* statistic in the two biological compartments. The slight negative shift of the villus compartment (relative to the crypts) indicates that a greater proportion of genes are expected to have an inverse relationship between the two network perturbations (e.g. when one network node increases in abundance in *Cdkn1a^-/-^*, the same node might decrease in *Apc^1638N+/-^* ).
