# Supplementary material for: Prediction and Testing of Biological Networks Underlying Intestinal Cancer
Source: PLoS One. 2010 Sep 1;5(9):e12497. doi: 10.1371/journal.pone.0012497 (PMC2931697; doi:10.1371/journal.pone.0012497)
Supplement: Methods S1 — Methods covering the statistical analysis of 2D-DIGE targets; the analysis of OMIM; and the construction of the filtered protein-protein interaction network. (0.08 MB DOC) [file pone.0012497.s002.doc]

**Supplementary Methods:**

**Table of Contents**

Page(s)

Methods: 2D-DIGE target significance testing

for the *Apc-Cdkn1a* network ………...………………………………………………….2

Methods: Analysis of OMIM…………………………………………………………..………….3

Methods: Forming a Filtered Protein-Protein Interaction Network………..……….….…….... 4-8

References………………………………………………………………………….….……... 9-10

**Supplementary Methods:**

**Significance Testing and Null Hypotheses for the *Apc-Cdkn1a* Network**

When aggregating *p*-values, the null hypothesis, *H_o_*, is that each component null, $H_{o, i}$, is true. A component null, $H_{o, i}$, can be stated as “network node, *i*, does not have a strong physical/coexpressive relationship with the DIGE targets from mouse mutant XYZ.” Thus, the four *H_o_* are as follows:

1. All network nodes do not have strong second-degree physical interactions with the *Apc^1638N+/-^* DIGE targets
2. All network nodes do not have strong second second-degree physical interactions with the *Cdkn1a^-/-^* DIGE targets
3. All network nodes are not coexpressed with the *Apc^1638N+/-^* DIGE targets
4. All network nodes are not coexpressed with the *Cdkn1a^-/-^* DIGE targets
5. All differentially expressed network nodes are not coexpressed with the *Apc^1638N+/-^* DIGE targets
6. All differentially expressed network nodes are not coexpressed with the *Cdkn1a^-/-^* DIGE targets

The *p*-values were aggregated as follows:

$$\tau=-2\ln\prod_{i\in G}^{n} p_{i}$$

Where *G* represents the set of proteins in the *Apc-Cdkn1a* network of size |*G*|=*n*; significance of τ is calculated from a$\chi^{2}$distribution with 2*n* degrees of freedom. The *p*-value of this aggregate measure, *τ*, is the probability of a $\chi_{2n}^{2}$ statistic being at least as large as *τ* if all the individual hypotheses, *H_o,i_*, are true. It was found that there was sufficient evidence to reject null hypotheses 1-3 and 5 at the *α*=1x10^-6^ significance level.

**Supplementary Methods:**

**Analysis of OMIM**

**Online Mendelian Inheritance in Man (OMIM)**

The list of oncogenes genes was compiled by searching the OMIM database for all genes whose titles or references contain the phrase “oncogene,” disregarding those entries whose molecular basis is unknown. As of Nov. 3, 2009, this resulted in 328 putative oncogenes, 9 of which are contained in the *Apc*-*Cdkn1a* signaling network. Significance was calculated using Fisher’s exact test.

**Supplementary Methods:**

**Forming a Filtered Protein-Protein Interaction Network**

**Eliminating False Positives via a logistic regression model**: Analysis based on the agreement of PPI and their expression data shows less than half of these interactions are biologically relevant [1]. In our logistic regression model, we incorporate (1) the number of times an interaction between two proteins was observed [2,3], (2) the Pearson correlation of expression measurements for the corresponding genes, (3) the proteins’ small world clustering coefficient, and (4) the protein subcellular localization data of interacting partners[4]. As expected, the protein subcellular localization data would eliminate interactions among proteins that are unlikely to come into contact with each other. Given the four input variables, $X=(X_{1},X_{2},X_{3},X_{4})$*,* the probability of a true interaction between two proteins $i$ and $j$, $\Pr(I_{ij})$ under the logistic distribution is $\Pr(I_{uv}|X)=\frac{1}{1+\exp(-\beta_{0}-\int_{i=1}^{4} \beta_{i}X_{i})}$. Given positive and negative training data sets, one can optimize the parameters to maximize the likelihood of a true interaction. We acquired randomly selected 1000 PPIs from the MIPS[5] database of interactions, an accepted gold standard as our positive data set. The negative training set was composed of 1000 randomly selected PPIs not contained in the MIPS database, since false positives are in high abundance in the training data sets (similarly employed in [3,6,7]). These experiments were repeated 1000 times and a cut off point for the probability of true interactions was determined.

**Recovering false negative interactions via homology****:** A protein family is a group of evolutionarily related proteins based on sequence similarity. Additionally, it has been also observed that sequence-wise similar proteins share similar interaction patterns in the same organism[8], suggesting that proteins within the same family are likely to have similar interaction patterns. The Protein Family database was downloaded from Pfam [9], and we inferred an edge between two proteins if (1) they are not already known to interact in the PPIN, and (2) there exists at least one interaction between the families of these two proteins.

**GO Annotations and Association Rule Mining:** Biological annotations, e.g., Gene Ontology [10] Biological Process (GO-BP) annotations provide a basis to find functionally similar proteins. We map proteins in both known signaling pathways and protein-protein interaction networks to their annotations. In this study, the training data of known signaling pathways is collected from various publicly available pathways databases [5,11,12,13], and pathway connections are converted to tuples of interacting proteins. Next, GO-BP annotations of pathway proteins are collected and kept as functionality sets. For each protein, associations between gene product and GO terms are queried from the GO Database; the Biological Process ontology terms acquired are leaf nodes on the directed acyclic GO term graph. Each annotation of a protein is linked with its interacting neighbor’s annotations and a network of annotation links is formed. All possible combinations are examined since they represent all possible functional associations.

Association rule mining is then used to discover rules of GO annotation pairs, collecting the underlying patterns of signaling pathways to form a library of templates. The data used in this study generated results with 28572 observed association rules when the support value is 0.000003 and the confidence is 0.001 (Refer to [7] on how to pick a threshold). These rules and parameters are used to evaluate candidate pathway segments for possible occurrences of these rules.

**Interactions with weight assignments****:** A *weighted* PPIN is formed by calculating Pearson correlation coefficient of the interacting pairs’ gene expression levels. In this study, the absolute value of *corr*(*e*) is used to capture correlation (*r*=|*corr*(*e*)|>0.7). Usually the correlation of the expression genes provides some evidence as to whether the produced proteins are biologically related.

**Searching for pathway segments:** Our hypothesis is that, given association rules that capture the characteristics of some known pathways, and a weighted PPIN, a pathway segment should belong to a pathway if (1) it contains at least a certain number of these rules and (2) the average weight of interactions is above a given threshold. For filtering by GO annotation association rules, each pair of interacting proteins’ functional annotations is checked for a match with a tuple from the association rules set (the “rules” being defined from *a priori* known signaling pathways, e.g. KEGG).

For each selected path, an average *absolute* expression correlation coefficient is also calculated, which is then compared to the threshold (|*r|*>0.7). This extra filtering improves the outcome since true interactions often exhibit stronger correlation with expression levels [14]. These values are more apparent when examining signaling paths, i.e. chains of proteins. The candidate paths with *p*-values higher than the threshold are returned as query results.

**References**

1. Deane CM, Salwinski L, Xenarios I, Eisenberg D (2002) Protein interactions: two methods for assessment of the reliability of high throughput observations. Mol Cell Proteomics 1: 349--356.

2. Deng M, Sun F, Chen T (2003) Assessment of the reliability of protein-protein interactions and protein function prediction. Pac Symp Biocomput. Molecular and Computational Biology Program, Department of Biological Sciences, University of Southern California, 1042 West 36th Place, Los Angeles, CA 90089-1113, USA. pp. 140--151.

3. Sharan R, Suthram S, Kelley RM, Kuhn T, McCuine S, et al. (2005) Conserved patterns of protein interaction in multiple species. Proc Natl Acad Sci U S A 102: 1974--1979.

4. Huh WK, Falvo JV, Gerke LC, Carroll AS, Howson RW, et al. (2003) Global analysis of protein localization in budding yeast. Nature 425: 686--691.

5. Mewes HW, Heumann K, Kaps A, Mayer K, Pfeiffer F, et al. (1999) MIPS: a database for genomes and protein sequences. Nuc Ac Res 27: 44--48.

6. Scott J, Ideker T, Karp RM, Sharan R (2006) Efficient algorithms for detecting signaling pathways in protein interaction networks. J Comput Biol 13: 133--144.

7. Bebek G (2007) Analyzing and Modeling Large Biological Networks: Inferring Signal Transduction Networks. Cleveland: Case Western Reserve University. 140 p.

8. Bebek G, Berenbrink P, Cooper C, Friedetzky T, Nadeau J, et al. (2006) The degree distribution of the generalized duplication model. Theoretical Computer Science 369: 239--249.

9. Finn RD, Tate J, Mistry J, Coggill PC, Sammut SJ, et al. (2008) The Pfam protein families database. Nucleic Acids Res 36: D281-288.

10. Ashburner M, Ball CA, Blake JA, Botstein D, Butler H, et al. (2000) Gene ontology: tool for the unification of biology. The Gene Ontology Consortium. Nat Genet 25: 25--29.

11. Campagne F, Neves S, Chang CW, Skrabanek L, Ram PT, et al. (2004) Quantitative information management for the biochemical computation of cellular networks. Sci STKE 2004.

12. Gough NR, Adler EM, Ray LB (2004) Focus Issue: Cell Signaling--Making New Connections. Sci STKE 2004: 12.

13. Kanehisa M, Araki M, Goto S, Hattori M, Hirakawa M, et al. (2008) KEGG for linking genomes to life and the environment. Nucleic Acids Res 36: D480-484.

14. Suthram S, Shlomi T, Ruppin E, Sharan R, Ideker T (2006) A direct comparison of protein interaction confidence assignment schemes. BMC Bioinformatics 7: 360+.
