# Supplementary material for: Prediction and Testing of Biological Networks Underlying Intestinal Cancer
Source: PLoS One. 2010 Sep 1;5(9):e12497. doi: 10.1371/journal.pone.0012497 (PMC2931697; doi:10.1371/journal.pone.0012497)
Supplement: Table S1 — Lists of 2D-DIGE targets identified in the intestinal crypts and villi of the Apc1638N+/− and Cdkn1a−/− mice. (0.12 MB DOC) [file pone.0012497.s003.doc]

Table S1. List of proteins found differentially expressed in the villi and crypts of *Apc^1638N^*^+/-^ mice (compared to the villi and crypts, respectively, of wild-type mice). P-values less than 0.05 are highlighted in yellow. Upregulation is highlighted in green; downregulation is highlighted in red.

| **Spot**  **#** | **Location** | **Gene Symbol** | **SwissProt Acc** | **Coverage (%)** | **Mass (kDa)** | **pI** | **Score** | **t-test**  **p-value** | **LIMMA**  **p-value** | **Fold**  **change** |
| --- | --- | --- | --- | --- | --- | --- | --- | --- | --- | --- |
| 442 | Villi | ALDH1L1 | Q8R0Y6 | 58 | 98647 | 5.64 | 3906 | 0.184 | 0.039 | 1.64 |
| 442 | Villi | VIL1 | Q62468 | 47 | 92743 | 5.72 | 4724 | 0.184 | 0.039 | 1.64 |
| 463 | Villi | VIL1 | Q62468 | 62 | 92743 | 5.72 | 8484 | 0.012 | 0.002 | 1.871 |
| 479 | Villi | EEF2 | P58252 | 51 | 95253 | 6.41 | 5624 | 0.001 | 0.004 | 1.893 |
| 665 | Villi | HSPA5 | P20029 | 64 | 72377 | 5.07 | 11781 | 0.09 | 0.051 | 0.627 |
| 692 | Villi | GPD2 | Q64521 | 58 | 80848 | 6.17 | 5756 | 0.027 |  | 1.403 |
| 782 | Villi | LCP1 | Q3V0K9 | 60 | 70363 | 7.21 | 6060 | 0.02 | 0.003 | 2.357 |
| 900 | Villi | EPHX2 | P34914 | 17 | 62475 | 5.85 | 1316 | 0.034 | 0.031 | 1.507 |
| 902 | Villi | HSPD1 | P63038 | 42 | 60917 | 5.91 | 2937 | 0.016 |  | 1.57 |
| 1026 | Villi | KRT8 | Q61463 | 80 | 54531 | 5.42 | 14772 | 0.055 | 0.009 | 0.414 |
| 1110 | Villi | KRT8 | P11679 | 55 | 53210 | 5.42 | 4713 | 0.076 | 0.038 | 0.571 |
| 1110 | Villi | HNRNPF | Q9Z2X1 | 51 | 45701 | 5.31 | 3244 | 0.076 | 0.038 | 0.571 |
| 1115 | Villi | ACTR3 | Q99JY9 | 68 | 47327 | 5.61 | 4497 | 0.006 | 0.012 | 1.703 |
| 1176 | Villi | OAT | P29758 | 71 | 48324 | 6.19 | 9578 | 0.039 |  | 1.515 |
| 1226 | Villi | NDUFS2 | Q91WD5 | 55 | 52592 | 6.52 | 3419 | 0.028 |  | 1.32 |
| 1226 | Villi | OAT | P29758 | 52 | 48324 | 6.19 | 3253 | 0.028 |  | 1.32 |
| 1375 | Villi | ACTB | P60710 | 49 | 41710 | 5.29 | 1567 | 0.015 | 0.011 | 0.594 |
| 1402 | Villi | BPNT1 | Q9Z0S1 | 78 | 33175 | 5.54 | 4154 | 0.024 | 0.016 | 1.781 |
| 1431 | Villi | GNA11 | P21278 | 55 | 41997 | 5.7 | 1949 | 0.059 | 0.007 | 1.857 |
| 1533 | Villi | GAPDH | P16858 | 64 | 35787 | 8.44 | 4484 | 0.055 | 0.008 | 1.913 |
| 1552 | Villi | GPD1 | P13707 | 50 | 37560 | 6.75 | 2198 | 0.002 | 0.032 | 1.588 |
| 1552 | Villi | ANXA2 | P07356 | 59 | 39839 | 5.96 | 2160 | 0.002 | 0.032 | 1.588 |
| 1552 | Villi | OTC | P11725 | 50 | 38652 | 7.55 | 3748 | 0.002 | 0.032 | 1.588 |
| 1554 | Villi | MDH2 | P08249 | 50 | 35589 | 8.93 | 1268 | 0.053 |  | 1.467 |
| 1554 | Villi | GAPDH | P04797 | 48 | 35760 | 8.44 | 1387 | 0.053 |  | 1.467 |
| 1623 | Villi | MDH1 | P14152 | 42 | 36454 | 6.16 | 1597 | 0 | 0.009 | 1.74 |
| 1689 | Villi | SULT1B1 | Q9QWG7 | 64 | 34905 | 6.33 | 3318 | 0.144 | 0.049 | 1.879 |
| 1793 | Villi | KRT18 | P05784 | 43 | 47509 | 5.22 | 3682 | 0.006 | 0.007 | 0.481 |
| 1885 | Villi | NDUFS8 | Q5M9P5 | 45 | 24023 | 5.89 | 1689 | 0.037 |  | 0.769 |
| 1902 | Villi | KRT19 | P19001 | 57 | 44515 | 5.28 | 17414 | 0.013 | 0.012 | 0.49 |
| 1943 | Villi | ATP5H | Q91YK9 | 65 | 18738 | 5.52 | 2127 | 0.001 | 0.018 | 0.597 |
| 1943 | Villi | KRT20 | Q9D312 | 44 | 49004 | 5.31 | 3791 | 0.001 | 0.018 | 0.597 |
| 2011 | Villi | KRT19 | P19001 | 60 | 44515 | 5.28 | 3948 | 0.005 |  | 0.426 |
| 442 | Crypt | ALDH1L1 | Q8R0Y6 | 58 | 98647 | 5.64 | 3906 | 0.029 |  | 0.639 |
| 442 | Crypt | VIL1 | Q62468 | 47 | 92743 | 5.72 | 4724 | 0.029 |  | 0.639 |
| 628 | Crypt | IMMT | Q8CAQ8 | 74 | 83848 | 6.18 | 9144 | 0.044 |  | 0.702 |
| 906 | Crypt | PDIA3 | P27773 | 63 | 56586 | 5.98 | 4423 | 0.043 | 0.019 | 1.534 |
| 930 | Crypt | KRT8 | P11679 | 16 | 54531 | 8 | 1117 | 0.009 | 0.015 | 1.625 |
| 1661 | Crypt | CAPZB | A2AMV7 | 71 | 33746 | 6.02 | 3020 | 0.165 | 0.044 | 1.804 |

Table S2. List of proteins found differentially expressed in the villi and crypts of *Cdkn1a*^-/-^ mice (compared to villi and crypts, respectively, of wild-type mice). P-values less than 0.05 are highlighted in yellow. Upregulation is highlighted in green; downregulation is highlighted in red.

| **Spot**  **#** | **Location** | **Gene**  **Symbol** | **SwissProt**  **ID** | **Coverage**  **(%)** | **Mass (kDa)** | **pI** | **Score** | **t-test**  **p-value** | **LIMMA**  **p-value** | **Fold**  **change** |
| --- | --- | --- | --- | --- | --- | --- | --- | --- | --- | --- |
| 1552 | Villi | ANXA2 | P07356 | 59 | 39839 | 5.96 | 2160 | 0.04 |  | 1.32 |
| 1039 | Villi | ATP5A1 | Q03265 | 49 | 59716 | 9.22 | 4277 | 0.09 | 0.05 | 1.50 |
| 479 | Villi | EEF2 | P58252 | 51 | 95253 | 6.41 | 5624 | 0.04 | 0.05 | 1.53 |
| 1039 | Villi | GLUD1 | Q8C273 | 61 | 61298 | 8.05 | 5415 | 0.09 | 0.05 | 1.50 |
| 1552 | Villi | GPD1 | P13707 | 50 | 37560 | 6.75 | 2198 | 0.04 |  | 1.32 |
| 1793 | Villi | KRT18 | P05784 | 43 | 47509 | 5.22 | 3682 | 0.04 |  | 0.65 |
| 2011 | Villi | KRT19 | P19001 | 60 | 44515 | 5.28 | 3948 | 0.01 |  | 0.43 |
| 1032 | Villi | KRT8 | Q3TJE1 | 83 | 54514 | 5.42 | 13024 | 0.04 |  | 0.68 |
| 1623 | Villi | MDH1 | P14152 | 42 | 36454 | 6.16 | 1597 | 0.04 |  | 1.30 |
| 1552 | Villi | OTC | P11725 | 50 | 38652 | 7.55 | 3748 | 0.04 |  | 1.32 |
| 463 | Villi | VIL1 | Q62468 | 62 | 92743 | 5.72 | 8484 | 0.01 | 0.02 | 1.50 |
| 691 | Crypts | HSPA9 | P38647 | 56 | 73483 | 5.91 | 7584 | 0.16 | 0.03 | 1.86 |
